# Enhancing the Performance of Dye Sensitized Solar Cells Using Silver Nanoparticles Modified Photoanode

**DOI:** 10.3390/molecules25174021

**Published:** 2020-09-03

**Authors:** Faizah Saadmim, Taseen Forhad, Ahmed Sikder, William Ghann, Meser M. Ali, Viji Sitther, A. J. Saleh Ahammad, Md. Abdus Subhan, Jamal Uddin

**Affiliations:** 1 Center for Nanotechnology, Department of Natural Sciences, Coppin State University, 2500 W. North Ave, Baltimore, MD 21216, USA; faizahsaadmim@gmail.com (F.S.); tforhad04@gmail.com (T.F.); asikder1@umbc.edu (A.S.); wghann@coppin.edu (W.G.); 2 Department of Neurosurgery, Cellular and Molecular Imaging Laboratory, Henry Ford Hospital, Detroit, MI 48202, USA; mali8@hfhs.org; 3 School of Computer, Morgan State University, Mathematical and Natural Sciences, Morgan State University, Baltimore, MD 21251, USA; viji.sitther@morgan.edu; 4 Department of Chemistry, Jagannath University, Dhaka 1100, Bangladesh; saleh203@yahoo.com; 5 Department of Chemistry, School of Physical Sciences, Shah Jalal University of Science and Technology, Sylhet 3114, Bangladesh; subhan-che@sust.edu

**Keywords:** dye sensitized solar cell (DSSC), silver nanoparticles (AgNPs), titanium dioxide (TiO_2_), electrochemical impedance spectroscopy (EIS), modified photoanode

## Abstract

In this study, silver nanoparticles were synthesized, characterized, and applied to a dye-sensitized solar cell (DSSC) to enhance the efficiency of solar cells. The synthesized silver nanoparticles were characterized with UV–Vis spectroscopy, dynamic light scattering, transmission electron microscopy, and field emission scanning electron microscopy. The silver nanoparticles infused titanium dioxide film was also characterized by Fourier transform infrared and Raman spectroscopy. The performance of DSSC fabricated with silver nanoparticle-modified photoanode was compared with that of a control group. The current and voltage characteristics of the devices as well as the electrochemical impedance measurements were also carried out to assess the performance of the fabricated solar cells. The solar-to-electric efficiency of silver nanoparticles based DSSC was 1.76%, which is quite remarkable compared to the 0.98% realized for DSSC fabricated without silver nanoparticles.

## 1. Introduction

There is an inescapable need for sustainable and cleaner energy all over the world and currently, the main source of energy in the world is fossil fuels like natural gas, coal, and oil. However, fossil fuels are not ideal energy sources as they perpetuate pollution, making their continuous use unsustainable and a cause of hazardous environmental problems [1,2]. Thus, the development of renewable energy is integral in addressing the growing demand for cleaner and more sustainable energy throughout the world [3,4]. The main sources of renewable energy that have been explored recently are solar, wind, hydro, biomass, and geothermal energy. In particular, solar energy already provides energy for all organisms on Earth through the process of photosynthesis [5,6,7]. Through the use of photovoltaic cells, sunlight can be converted into and utilized as electricity [8,9,10]. Since solar energy is the most abundant form of renewable energy, solar cells create the possibility of easily accessible energy for inhabitants in various regions throughout the world.

Current widely available solar cells are based on inorganic materials, require expensive methods of production, and make use of a p-n/heterojunction and other metal-semiconductor junctions. However, dye-sensitized solar cells (DSSC) provide a feasible alternative to present-day inorganic p-n junction solar cells by converting solar energy into electric energy through the photosensitization of the cell [11,12,13]. Compared to solar cells based on inorganic materials, DSSCs require relatively low costs, involve an easier method of production, and are environmentally sustainable [14,15]. Nonetheless, DSSCs result in compromised efficiencies and its longstanding durability is in question. The efficiency of the DSSCs in converting solar energy can be increased by manipulating parts of the cell, for instance, the cathode or adding nanoparticles like silver (Ag) to enhance its performance [16]. The use of nanoparticles, especially silver and gold nanoparticles in DSSCs in an effort to enhance the solar-to-electric power efficiency, creates what is termed as the plasmonic effect [17]. When light interacts with the free electrons (surface plasmons) of the metal nanoparticles, surface plasmon resonance (SPR), a property of metal nanoparticles, is created. This results in the oscillations of the surface plasmons, creating an extinction of light which increases the light absorption of surrounding dye molecules. These plasmonic nanoparticles enhance the light absorption properties of the sensitizing dye and also improve the electron dynamics. Incorporating Silver Nanoparticles (AgNPs) increases light absorption in the photo-anode layer of DSSCs. The use of plasmonic nanoparticles (NPs) in DSSCs not only boosts their power conversion efficiencies (PCEs) by increasing light absorbance of the cells, but also affects their electron dynamics.

In this work, the DSSCs composed of AgNP-modified photoanodes were fabricated and characterized. The performances of DSSC with and without AgNPs were compared and there was found to be improved efficiency for DSSCs with AgNPs.

## 2. Experimental Section

### 2.1. Materials and Instrumentation

Fluorine-doped tin oxide (FTO) conducting glass slides were purchased from Harford Glass Company, Hartford City, Indiana, USA. Sodium hydroxide (NaOH), acetone (C_3_H_6_O), ethanol (C_2_H_5_OH), and acetic acid (CH_3_CO_2_H) were purchased from Sigma-Aldrich (St. Louis, MS, USA) and were used without further purification. The synthetic N719 dye and silver nitrate (AgNO_3_) were bought from Fisher Scientific and used in its original form. Colloidal graphite used to prepare the counter electrode was purchased from Ted Pella Inc, Redding, CA 96003, USA. Titanium dioxide powder (Degussa P-25) was purchased from the Institute of Chemical Education (University of Wisconsin-Madison, Department of Chemistry, Madison, WI 53706, USA). The morphology of synthesized AgNPs was analyzed using field emission scanning electron microscopy (Model FESEM: JSM-7100FA JEOL USA, Inc.). The AgNPs were further characterized by high-resolution transmission electron microscope (HRTEM), JEM-1400 PLUS (JEOL USA, Peabody, MA, USA). The images were viewed using digital micrograph software from GATAN (GATAN Inc., Pleasanton, CA 94588, USA). Absorption spectroscopy was carried out with a UV-3600 Plus from Shimadzu, Columbia, MD 21046, USA. Emission spectroscopy was measured with a RF-5301PC from Shimadzu, Columbia, MD 21046, USA. Raman studies were carried with a model DXR smart Raman spectrometer (Thermo Fisher Scientific Co. Ltd., Waltham, MA 02451, USA). ATR spectra were obtained with a Thermo Nicolet iS50 FTIR. TiO_2_ paste was printed on FTO glass using a WS-650 Series Spin Processor from Laurell Technologies Corporation, North Wales, PA 19454, USA. The cell performance was measured using a 150 W fully reflective solar simulator with a standard illumination of air-mass 1.5 global (AM 1.5 G) having an irradiance of 100 mW/cm^2^ (Sciencetech Inc. London, ON N6N 1R3, Canada). Reference 600 Potentiostat/Galvanostat/ZRA used for current, voltage, and impedance measurements was purchased from GAMRY Instruments (Warminster, PA 18974, USA).

### 2.2. Synthesis of Silver Nanoparticles

Silver Nanoparticles (AgNPs) were prepared by adding 1 mM of silver nitrate (AgNO_3_) to 2 mM sodium borohydride (NaBH_4_), the reducing agent, and heating for 10 min to form a uniform solution. The 1 mM of AgNO_3_ was added drop-wise, approximately one drop per second, to the borohydride solution, and after 10 min of heating at a temperature of 70 °C under constant stirring with a magnetic stir rod at 100 rpm, the solution was removed for cooling. Consequently, the color of the solution turned yellow as a result of SPR, indicating the formation of AgNPs. The shade of yellow of the solution served as an early indicator of the size of the AgNPs.

### 2.3. Fabrication of Dye-Sensitized Solar Cell

Preparation of the AgNPs-infused titanium dioxide films was carried out with the AgNPs synthesized from sodium borohydride and silver nitrate as the reducing agent. The photoanode was prepared by depositing a thin film of TiO_2_ infused with AgNPs on the conductive side of a FTO glass slide using a spin coater and annealing the film at 380 °C for an hour. The titanium dioxide paste was prepared with 3 mL silver nanoparticle solution, 3 mL acetic acid, 1 mL of dish soap water, and 2.4 g of titanium dioxide powder. For samples with titanium tetrachloride (TiCl_4_), the TiO_2_ coated FTO glass was subsequently dipped into the TiCl_4_ solution at 70 °C for an hour and annealed again for 30 min at 450 °C. The substrate was then immersed overnight in a prepared N719 dye solution. The thickness of the titanium dioxide film on the FTO glass was determined by field emission scanning microscopy cross-sectional imaging to be 8 µm. The thickness of the titanium dioxide film determines its dye loading capacity and has been shown to influence the efficiency of the dye-sensitized solar cell. Subramanian et al. reported the loading capacity of P25 with a thickness of 13 µm to be 2.4 µmol/cm^2^ [18]. The counter electrode (cathode) was prepared using colloidal graphite. The FTO glass was first cleaned with water and ethanol and the colloidal graphite plastered uniformly on the conductive side of the FTO glass. The device was finally assembled according to a protocol already published [19]. The dye-sensitized photoanodes and the carbon electrodes were combined to form a solar cell by sandwiching them with a redox iodine/iodide electrolyte solution. The electrolyte solution was composed of iodine (I_2_) and potassium iodide (KI) in ethylene glycol. The electrolyte was dropped between the photoanode and counter electrode and allowed to spread down by capillary.

## 3. Results and Discussion

### 3.1. Absorption Measurements

**Figure 1:**
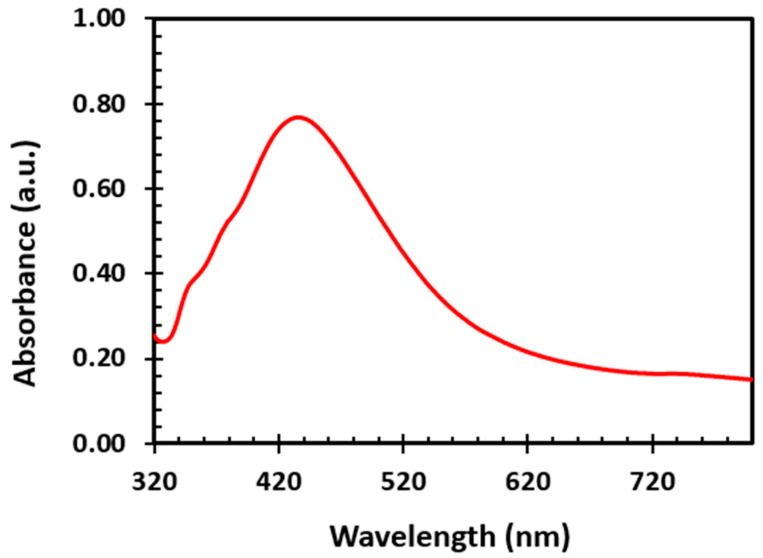
Absorption Spectra of Silver Nanoparticles (AgNPs) showing the SPR band.

UV–Visible absorption spectroscopy was carried out to characterize the absorption of the Silver Nanoparticles (AgNP) solution. The UV–Vis spectra of the synthesized aqueous silver nanoparticles is shown in Figure 1. The wavelength of maximum absorption for the AgNPs was 436 nm. The absorption band at 436 nm is indicative of the surface plasmon resonance (SPR) band of silver nanoparticles and thus the formation of silver nanoparticles. The SPR results from the interaction of free electrons and electromagnetic radiation. This phenomenon enhances the absorption coefficient of the dye and optical absorption, which results in the increase in the efficiency of the solar cell [20,21,22,23].

### 3.2. Dynamic Light Scattering Measurements

**Figure 2:**
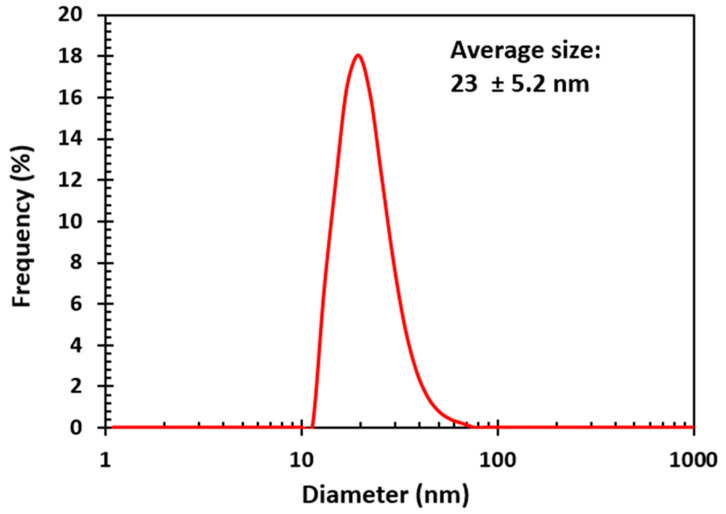
Dynamic light scattering measurement of synthesized silver nanoparticles.

Dynamic light scattering (DLS) was used to measure the size of nanoparticles and their dispersity in solution. Thus, one can obtain the hydrodynamic diameter as well as the size distribution curve of the sample. The silver nanoparticle synthesized for application in DSSC was characterized with DLS and the results of the measurements are displayed in Figure 2. The average size of the AgNPs was 23 nm with a standard deviation of 5.2 nm. The hydrodynamic diameter takes into the consideration the nanoparticle core size as well as the shell of water around the nanoparticles [24].

### 3.3. Field Emission Scanning Electron Microscopy Imaging and Energy Dispersive X-ray Spectroscopy

**Figure 3:**
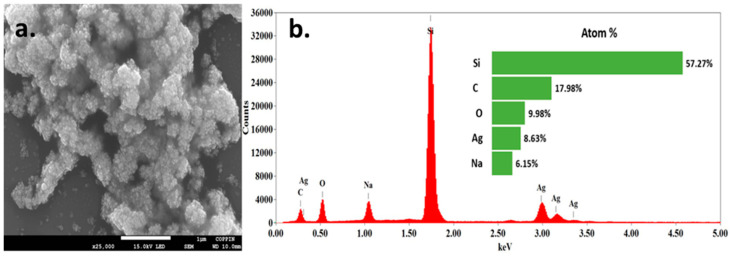
Field emission scanning electron microscopy imaging (**a**) and the corresponding energy dispersive X-ray spectroscopy (**b**) of the synthesized silver nanoparticles.

The synthesized silver nanoparticles were further characterized by field emission scanning electron microscopy (FESEM) imaging to obtain morphological information and with energy dispersive X-ray spectroscopy (EDS) for elemental analysis of the silver nanoparticles sample. The results of the measurements are illustrated in Figure 3. The FESEM image in Figure 3a shows that the particles were nearly spherical in shape and turned to agglomerate. The EDS spectrum in Figure 3b shows the presence of silicon, carbon, oxygen, silver, and sodium. The silicon peak represents the silver wafer substrate use in the imaging. The sodium likely originated from the sodium borohydride used in the reduction of silver nitrate to obtain the silver nanoparticles.

### 3.4. Transmission Electron Microscopy Imaging

**Figure 4:**
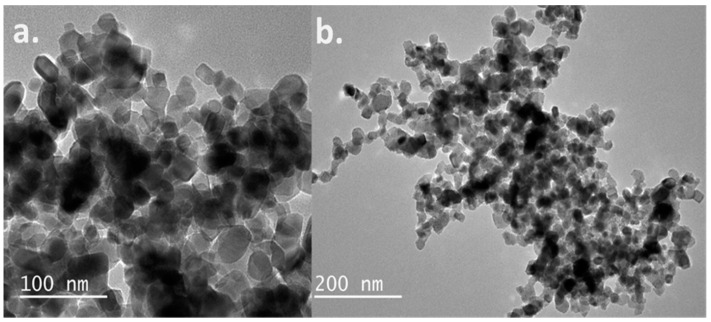
Transmission electron microscopy of Silver Nanoparticles (AgNPs) (**a**) and AgNPs with titanium dioxide nanoparticles (**b**).

The AgNPs as well as the AgNP infused titanium dioxide were characterized by transmission electron microscopy (TEM). The high resolution of the TEM allows for the visualization of the individual nanoparticles. The images of the analysis are displayed in Figure 4. The TEM image of the silver nanoparticles with titanium dioxide showed a close interaction of AgNPs with titanium dioxide. The sizes of the AgNPs and titanium dioxide were similar, which accounted for the excellent interaction between the nanoparticles.

### 3.5. Fourier Transform Infrared Measurements

**Figure 5:**
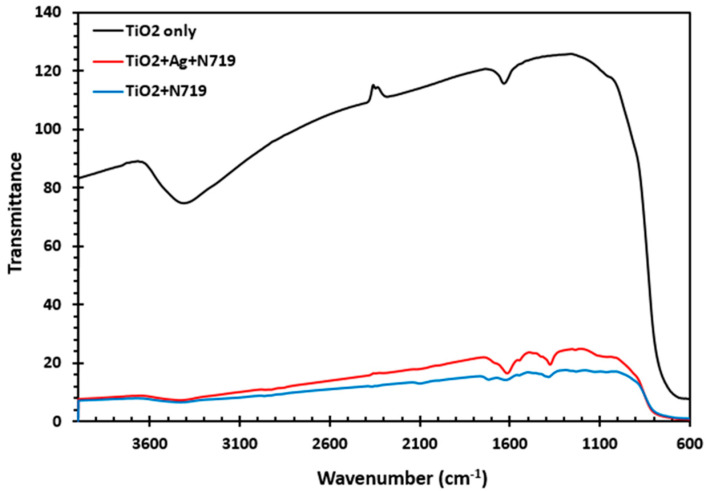
FTIR spectra of Silver Nanoparticles (AgNPs) infused titanium dioxide and bare titanium dioxide.

Fourier transform infrared (FTIR) measurements were performed on the titanium dioxide film and dye-sensitized titanium dioxide and silver nanoparticle-modified dye-sensitized titanium dioxide film to evaluate the N719 dye/AgNP/titanium dioxide interaction. The results of the FTIR measurements are shown in Figure 5. The IR spectra of the titanium dioxide film, as shown in Figure 5, show vibrational modes at 3413 cm^−1^ and 1622 cm^−1^, which were ascribed to TiO_2_ for υO-H at 3413 cm^−1^ and δO-H at 1622 cm^−1^, respectively. Peaks at 1463 cm^−1^ and 1627 cm^−1^ in both the dye-sensitized titanium dioxide and silver nanoparticle-modified dye-sensitized titanium dioxide film originated from the N719 dye. The band between 600 cm^−1^ and 700 cm^−1^ was attributed to the bending and stretching mode of Ti–O–Ti. The broadband in the region of around 3500–300 cm^−1^ was also a characteristic carboxyl group (O–H stretch) of the N719. The carboxyl group bond with the hydroxyl of the titanium dioxide providing a channel for the transfer of electrons.

### 3.6. Raman Spectroscopy

**Figure 6:**
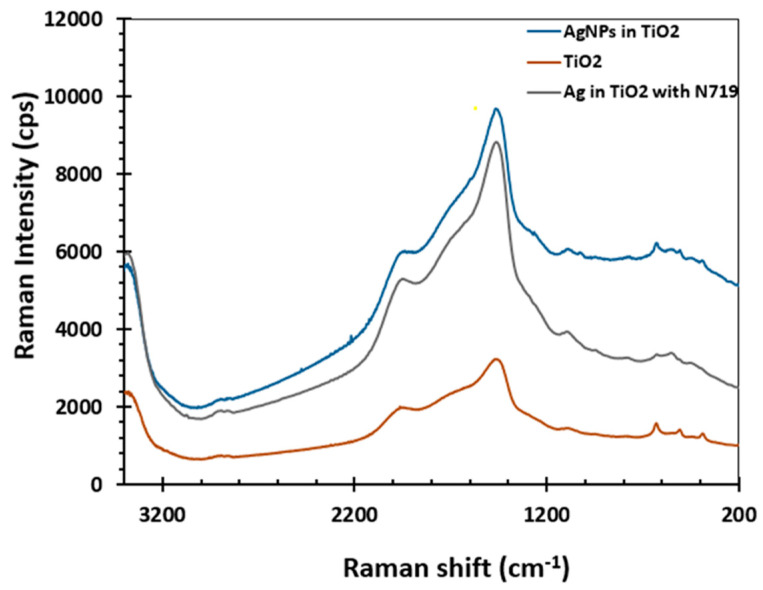
Raman spectra of Silver Nanoparticles (AgNPs) infused titanium dioxide and bare titanium dioxide.

The interaction between the AgNPs and the titanium dioxide was further evaluated with Raman spectroscopy. Raman studies on the silver doped dye-sensitized titanium dioxide films were performed in the range of 200–3400 cm^−1^, and the results are shown in Figure 6. The D and G bands at 1490 cm^−1^ and 1950 cm^−1^ respectively, are consistent with previous studies that attribute the peaks to the high disorder exhibited by sp^3^ carbons [25]. The presence of the additional peaks is an indication of the interaction between the TiO_2_ nanoparticles and the AgNPs.

### 3.7. Current and Voltage Characteristics

**Figure 7:**
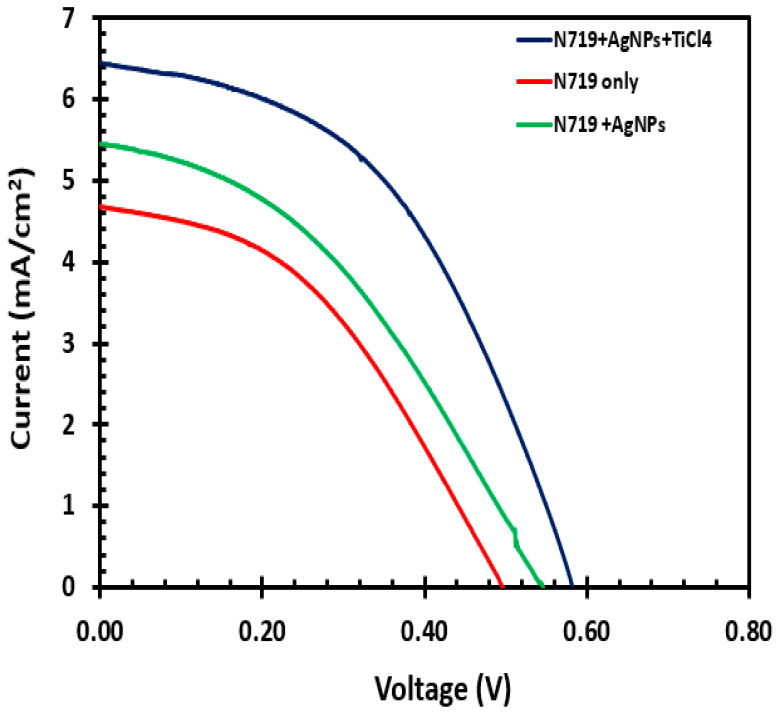
Current and voltage graph of DSSCs fabricated with only N719, N719, with AgNPs treatment, and N719 with AgNPs and TiCl_4_ treatment.

The performance of the DSSC was evaluated via the measurements of the open-circuit voltage, short-circuit current, fill factor, maximum voltage, and the maximum current of the cell. The current and voltage characteristic measurements of the fabricated solar cell are displayed in Table 1 and Figure 7. The DSSC fabricated with AgNPs and soaked in the TiCl_4_ solution gave the highest solar-to-electric power of efficiency of 1.76%, with an open-circuit voltage of 0.50 V, short-circuit current of 6.45 mA/cm^2^, and a fill factor of 0.47. The efficiency of N719 + TiO_2_ and N719 was 1.20% and 0.98%, respectively. The increase from 0.98% to 1.2% to a large extent was the result of the increase in the short circuit current from 4.69 mA/cm^2^ to 5.46 mA/cm^2^ as well as an increase in the open-circuit voltage. The fill factor decreased from 0.42 to 0.40. This could be due to resistance and leaks in the circuit. The overall enhanced photovoltaic performance could be attributed to the plasmonic effect of the AgNPs that result in a swift transfer of an electron from the AgNPs to the TiO_2_ [26]. The free electrons in metals can be excited by electrical components of light to produce collective oscillations. These oscillations could be confined in a very small volume around a silver nanoparticle, which results in enhancement of the local field and helps increase light incident on the nanoparticles. Thus, the silver nanoparticles embedded in the titanium dioxide improved the absorption of the light exposed to the dye sensitized solar cell and thus enhanced the efficiency of the solar cell. The use of the TiCl_4_ also provided an additional enhancement of the efficiency of the DSSC since the TiCl_4_ increased the dye adsorption [27]. The TiCl_4_ boosted the efficiency by increasing the dye absorption of the titanium dioxide, which consequently increased the short-circuit current density. There was a remarkable increase in the open-circuit voltage and an improvement in the fill factor. 

### 3.8. Electrochemical Impedance Spectroscopic Measurements

**Figure 8:**
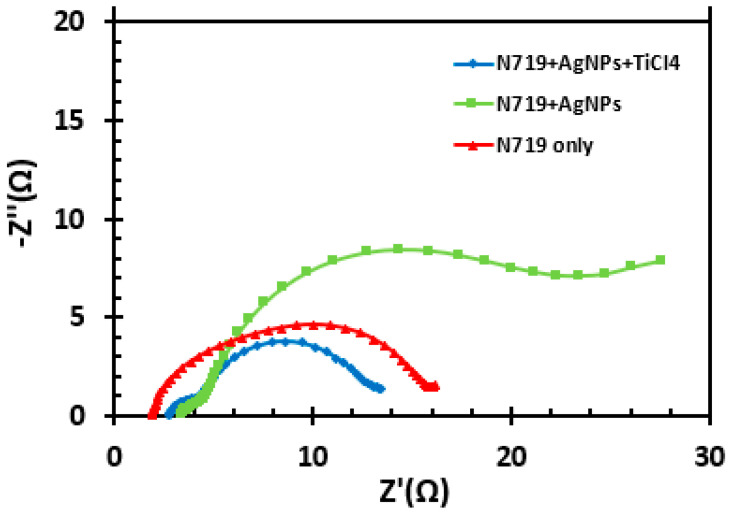
Nyquist plot of DSSCs fabricated with only N719, N719 with AgNPs treatment, and N719 with AgNP and TiCl_4_ treatment.

**Figure 9:**
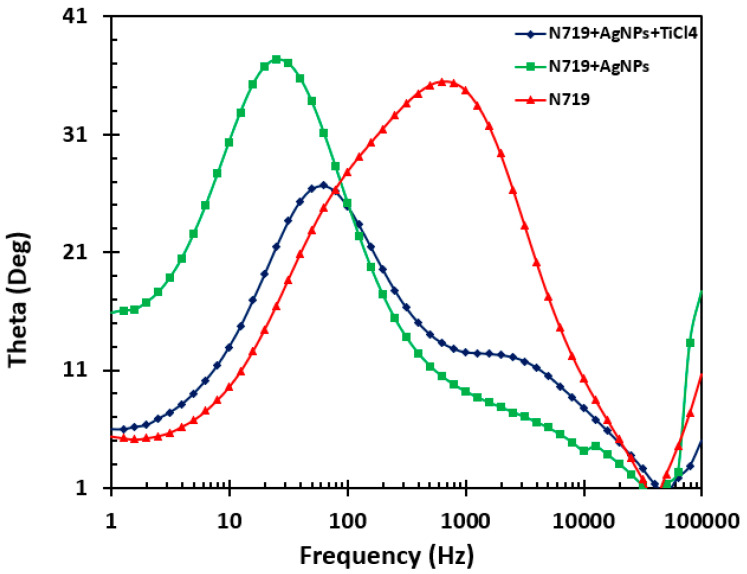
Bode plot of DSSCs fabricated with only N719, N719 with AgNPs treatment, and N719 with AgNPs and TiCl_4_ treatment.

Electrochemical impedance spectroscopic (EIS) measurements were performed to evaluate the charge transfer and resistance in the fabricated DSSC [28,29,30]. EIS data were presented in Nyquist and Bode plots, as displayed in Figure 8 and Figure 9, respectively. The measurements were performed under illumination.

A Nyquist plot typically displays two or three semicircles. A smaller semicircle at a high frequency corresponds to a charge transfer resistance at the cathode/electrolyte interface, while a larger semicircle corresponds to a charge transfer resistance at the titanium dioxide/dye/electrolyte interface.

The Bode plot correlates peak frequencies to the lifetime (τ) of the charge. The lifetime is inversely proportional to the peak frequency, as displayed in Equation (1).

τ = 1/2πf(1)

The Bode plot of the solar cell fabricated with AgNPs, as shown in Figure 9, has a lower frequency, thus leading to a relatively longer electron lifetime, which ultimately leads to the higher efficiency of the DSSC. A long electron lifetime is beneficial for the DSSC device as it will improve the open-circuit current from which the efficiency of the cell is calculated.

## 4. Conclusions

In this study, the AgNPs were synthesized, characterized, and applied in DSSC to enhance the efficiency of the solar cells. The performance of the DSSC fabricated using the N719 dye and AgNPs-modified paste of TiO_2_ was compared with that of a control group created using just the N719 dye. The silver and the pre-treatment of TiCl_4_ appeared to have the maximum effect on efficiency (1.76%). The enhanced photovoltaic performance of the DSSCs could be attributed to the plasmonic effect of the AgNPs and the use of TiCl_4_ that allowed for increased dye adsorption and a swift transfer of charge. The silver nanoparticles embedded in the titanium dioxide thus improved the absorption of the light exposed to the dye-sensitized solar cell and enhanced the efficiency of the solar cell.

## Figures and Tables

**Table 1:** Photovoltaic performance for fabricated dye-sensitized solar cell (DSSCs) with and without Silver Nanoparticles (AgNPs).

	Voc (V)	Jsc (mA/cm^2^)	Vmp (V)	Imp (mA/cm^2^)	Fill Factor	Efficiency (%)
N719	0.50	4.69	0.27	3.42	0.42	0.98
N719 + AgNPs	0.55	5.46	0.31	3.78	0.40	1.20
N719 + AgNPs + TiCl_4_	0.58	6.45	0.38	4.68	0.47	1.76
